# Advances in Topological Materials: Fundamentals, Challenges and Outlook

**DOI:** 10.3390/nano12193522

**Published:** 2022-10-08

**Authors:** Sławomir P. Łepkowski

**Affiliations:** Institute of High Pressure Physics—Unipress, Polish Academy of Sciences, ul. Sokołowska 29/37, 01-142 Warszawa, Poland; slawek@mail.unipress.waw.pl

The discovery of topological insulators, characterized by an energy gap in bulk electronic band structures and metallic states on boundaries, has greatly inspired studies on the topological properties of the electronic band structures of crystalline materials. Thanks to these studies, the Dirac and Weyl semimetals with topologically protected, linearly dispersing bands in bulk band structures have joined the family of topological materials. Recently, higher-order topological insulators—in which the gapless boundary states with dimensionality lower by two (or more) than the dimensionality of the bulk appear—have been discovered.

This Special Issue, containing eight research works and one review, is an interesting collection of papers on topological materials. The papers present a variety of materials including conventional 3D topological insulators such as HgTe, Bi_2_Se_3_, and (Bi_1−*x*_Sb*_x_*)_2_Te_3_ [1,2,3]; 3D antiferromagnetic topological insulator MnBi_2_Te_4_ [4]; 2D topological insulator in InN/InGaN quantum wells [5]; Weyl semimetals [6,7]; Floquet second-order topological phases realized in cold atom systems [8]. One paper is devoted to the quantum phase transition in Co-based magnetic tunnel junctions [9]. The studies presented in [1,2,3,4] are experimental, while [5,6,7,8,9] are theoretical. Here, we provide a brief overview of the individual papers.

We begin the discussion of the articles with a paper on the thermoelectric transport properties of a HgTe topological insulator by Gusev et al. [1]. This work presents an experimental study of thermopower as a function of the Fermi energy in strained HgTe films. The authors demonstrate that the thermopower changes significantly when the Fermi level crosses regions featuring the coexistence of 2D surface electrons and 3D bulk holes. The authors attribute this effect to the mutual scattering between carriers. The review paper [2] by Gracia-Abad et al. presents the quantum transport phenomena in a Bi_2_Se_3_ topological insulator, paying attention to the magnetotransport properties of these materials with particular emphasis on the weak-antilocalization effect. The authors carefully describe the different situations found in the reported experiments performed on Bi_2_Se_3_ thin films; the discussion included the most ideal situation, with a dominant contribution of the topologically protected surface states, and more realistic cases in which the presence of defects enhances the contribution of the bulk states; finally, they account for a situation where the bulk states completely dominate the electronic transport in these materials. The above cases are reflected in the dependences of the carrier mobility and the phase coherence length on the thickness of the layers. The growth of a (Bi_0.4_Sb_0.6_)_2_Te_3_ topological insulator using molecular beam epitaxy is investigated by Mulder et al. [3]. Their study reveals the substantial influence of the substrate on the formation of defects, mosaicity, and thin domains, which leads to the conclusion that (Bi_0.4_Sb_0.6_)_2_Te_3_ thin films grow by quasi-van der Waals epitaxy when the lattice mismatch between layers and the substrate is large. Su et al. report on the synthesis of MnBi_2_Te_4_ films having a single phase and high structural quality [4]. Their paper demonstrates that these materials possess topologically protected Dirac-like surface states in the paramagnetic phase at 80 K and an antiferromagnetic order at Neel temperature 22 K.

The search for new topological materials is one of the leading developments in solid-state physics. Łepkowski and Anwar demonstrate that a 2D topological insulator can occur in ultra-thin double quantum wells made of group-III nitride semiconductors [5]. This article presents the topological and nontopological phase transitions in In*_x_*Ga_1−*x*_N/GaN and InN/In*_y_*Ga_1−*y*_N double quantum wells and proposes a structure for which the bulk energy gap in the topological insulator state is the largest. The paper by Huang et al. reports that TlCd_2_Te_4_ alloys exhibit exotic states such as threefold fermions and nodal-line fermions which split into Weyl fermions when the spin–orbit coupling is included [6]. This work also reveals that electron-doped TlCd_2_Te_4_ can become a superconductor at low temperatures, allowing for the coexistence of the topological Weyl semimetal and superconductivity in a single material. Bonilla et al. present a study of electronic and thermoelectric transport in the Weyl semimetal with a torsional dislocation defect, in the presence of an external magnetic field parallel to the dislocation axis [7]. This article demonstrates that, in this system, the electric current exhibits chiral valley polarization, the conductance shows the signature of Landau levels, and the thermal transport coefficients—such as the thermopower and the figure of merit—reach large values. Zhou [8] demonstrates the second-order topological phases in time-periodic-driven systems, called the Floquet systems, which can be realized by cold atoms in optical lattices. These Floquet topological phases are protected by chiral symmetry and are characterized by the topological invariants which determine the number of the corner states via the bulk–corner correspondence. Finally, the report by Hussien and Ukpong [9] reveals an electric field induced quantum phase transition from the half-metallic to metallic phase in a symmetric Co-based magnetic tunnel junction with a monolayer hexagonal BN tunnel barrier. This effect does not occur when the tunnel barrier is made from monolayer MoS_2_ which shows that the choice of the tunnel barrier material plays an important role in determining the magnetoelectric coupling effect in these structures.
